# Autophagy, autophagy-associated adaptive immune responses and its role in hematologic malignancies

**DOI:** 10.18632/oncotarget.13583

**Published:** 2016-11-25

**Authors:** Liangshun You, Shenhe Jin, Li Zhu, Wenbin Qian

**Affiliations:** ^1^ Department of Hematology, The First Affiliated Hospital, College of Medicine, Zhejiang University, Hangzhou 310003, P.R. China

**Keywords:** autophagy, immune, adaptive immune, cancer immunotherapy, hematologic malignancy

## Abstract

Autophagy is a tightly regulated catabolic process that leads to the degradation of cytoplasmatic components such as aggregated/misfolded proteins and organelles through the lysosomal machinery. Recent studies suggest that autophagy plays such a role in the context of the anti-tumor immune response, make it an attractive target for cancer immunotherapy. Defective autophagy in hematopoietic stem cells may contribute to the development of hematologic malignancies, including leukemia, myelodysplastic syndrome, and lymphoproliferative disorder. In blood cancer cells, autophagy can either result in chemoresistance or induce autophagic cell death that may act as immunogenic. Based on the successful experimental findings *in vitro* and *in vivo*, clinical trials of autophagy inhibitor such as hydroxychloroquine in combination with chemotherapy in patients with blood cancers are currently underway. However, autophagy inactivation might impair autophagy-triggered anticancer immunity, whereas induction of autophagy might become an effective immunotherapy. These aspects are discussed in this review together with a brief introduction to the autophagic molecular machinery and its roles in hematologic malignancies.

## INTRODUCTION

Cell homeostasis is dependent on the balance between biosynthesis and catabolism of macromolecules. Eukaryotic cells possess two major protein degradation routes: the ubiquitin-proteasome and the lysosomal systems [1]. The proteasome system is responsible for the selective degradation of most short-lived proteins [2], while the lysosomal system degrades and recycles long-lived proteins and defective organelles, these substances from both inside and outside of cell are delivered to the lytic compartments [2, 3]. There are four major pathways to the lysosome for degradation: endocytosis/phagocytosis, microautophagy, chaperone-mediated autophagy (CMA), and macroautophagy [4, 5]. Degradation of exogenous materials and membrane proteins is mediated by the process of endocytosis/phagocytosis, whereas degradation of cytoplasmic component is carried out by microautophagy, CAM and macroautophagy [6].

**Figure 1:**
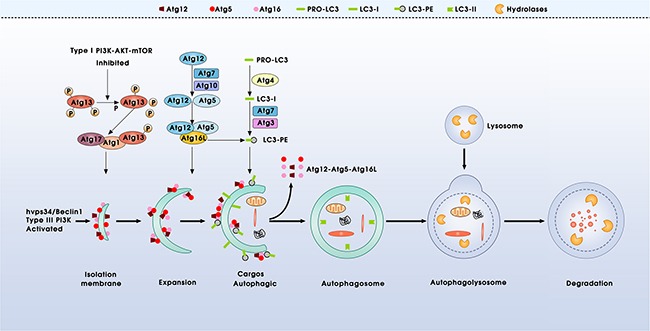
Schematic model of autophagy process Autophagy is a multi-step process involving at least four main phases, which is controlled by more than 30 autophagy-related (Atg) proteins and mediated by two ubiquitin-like conjugation systems, Atg12-Atg5 and Atg8/LC3: including the initiation, vesicle expansion and completion, maturation and fusion, and ultimate degradation of the membrane and its contents within the lysosomes.

Macroautophagy (hereafter referred to as “autophagy”) is the main form of autophagy, which is a multi-step process involving at least four stages [7, 8]: (i) autophagy induction: when cells were under the condition of stimulation of autophagy, the type I PI3K-AKT-mTOR signaling is inhibited and type III PI3K mammalian vps34/Beclin-1 (Atg6) is activated. Inhibition of mTOR reassociates dephosphorylated Atg13 with Atg1, which in turn results in redistribution of mAtg9 from trans-Golgi to late endosome and induces autophagy [9, 10]. Simultaneously, the activation of vps34/Beclin-1 generates phosphatidylinositol (3,4,5) P3 (PIP3) on endomembrane, resulting in isolation and decoration with Atg5 and Atg16 of a small template membrane, which designated as phagophore [11]. (ii) vesicle expansion and completion: the structure of phagophore could not get further progression without two ubiquitin-like conjugation systems. One pathway involves the covalent conjugation of Atg12 to Atg5, with the help of Atg7 (E1-like enzyme) and Atg10 (E2-like enzyme). Atg12-Atg5 successionally binds to Atg16 and multi-dimerizes to form a large complex [12–14]. The second pathway involves the conjugation of phosphatidyl ethanolamine (PE) to microtubule-associated protein1 light chain LC3 (homologue of mammalian Atg8) by the sequential action of Atg4, Atg7 and Atg3. Briefly, LC3 is cleaved by Atg4 to produce the cytosolic form LC3-I (non-lipidated, 18KD), which is activated by Atg7 and transferred to Atg3, then modified into autophagic-vesicle-associated form LC3-II (PE-conjugated, 16KD), used as a marker of autophagy [14, 15]. (iii) maturation and fusion: autophagosomes undergo maturation (including the encapsulation of cellular components), and then fuse with lysosomes to become autolysosomes. (iv) degradation: in the autolysosomes, engulfed components are eventually degraded by lysosomal enzymes (Figure 1).

Autophagy is involved in various aspects of biological processes, including cell survival/death, proliferation, differentiation, senescence, and carcinogenesis [16, 17]. The role of autophagy in cancer is controversial. The degradation mechanism enables cells to recycle cytoplasmic constituents and restore metabolic homeostasis, maintaining cells survival under harsh conditions. However, excessive autophagy also induces a non-apoptotic form of programmed cell death, termed as type II programmed cell death [18]. Not surprisingly, aberrant regulation of autophagy is associated with many diseases such as cancer, neurodegenerative disorders, myopathies, cardiovascular diseases and so on. Recently, a series of studies reveal a crucial role of autophagy pathway and its interacting proteins in the regulation of immune response. This review article will focus on autophagy-associated adaptive immune responses and its role in hematologic malignancies because a deeper understanding of the effects of autophagy on immune and autophagy-associated adaptive immune responses allow us to explore potential immunotherapeutic approaches to cure hematologic malignancies.

## AUTOPHAGY REGULATIONG SIGNALING PATHWAYS

**Figure 2:**
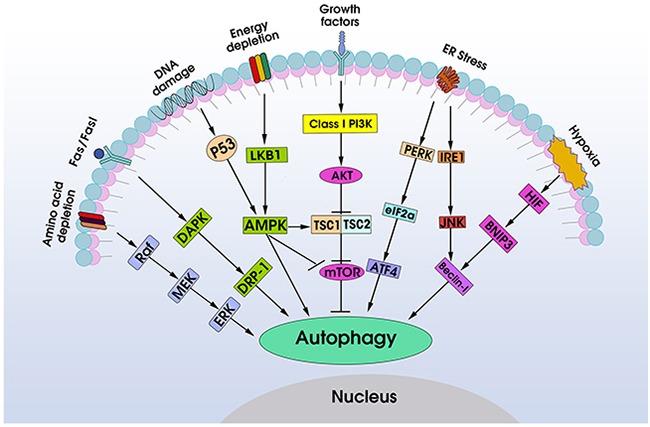
Schematic overview of autophagy-associated signaling pathways in cancer Autophagy can be activated under multiple stress situations during cancer progression, including amino acid depletion, hyper-expression of death-associated protein kinase, DNA damage, energy depletion, endoplasmic reticulum (ER) stress, hypoxia and other diverse stresses. The key signaling molecules are the PI3K-AMPK-mTOR signaling pathways, which determinate the levels of autophagy in cancer. Other autophagy-associated signaling pathways, including DAPK-DRP1, PERK-eIF2α-ATF4, IRE1-ASK1-JNK1-Beclin-1, p53/LKB1-AMPK, Raf-MEK-ERK, HIF1-BNPI3-Beclin-1 pathways, which are activated by one or more stresses, also play an important role in regulation of autophagy in cancer.

It is well known that autophagy is regulated by many signaling pathways (Figure 2). The PI3K- AMPK-mTOR signaling pathways play a central role in the regulation of autophagy [19, 20]. Among them, class I PI3Ks phosphorylatePI(4)P and PI(4,5)P2, which bind to the pleckstrin homology domain of AKT and its activator 3-phosphoinositide-dependent protein kinase-1 (PDK-1), as a result, activation of AKT attenuates autophagy [21–24]. Conversely, a dominant negative form of AKT enhances autophagy. AKT-PDK-1 signaling pathway activates a series of downstream signals, including mTOR that has been considered as a “gate keeper” of the autophagic pathway for it is a sensor for amino acids and ATP, two metabolites known to regulate autophagy [20, 25, 26]. In contrast to class I PI3Ks, the class III PI3Ks stimulate autophagy, which phosphorylate PI to generate PIP3 and participate in sequestration of cytoplasmic material in autophagic vacuoles [27]. An integral protein in the class III PI3Ks pathway is Beclin-1, a mammalian ortholog of yeast Atg6/Vps30, which is required for autophagosome formation. Knockdown or antisense Beclin-1 inhibits autophagy [28, 29]. The AMP-dependent protein kinase (AMPK) is activated during hypoxia, metabolic stress or ATP consumption by increased ratios of AMP to ATP and stimulates autophagy through inhibiting mTOR pathway [30, 31].

Endoplasmic reticulum (ER) stress response can induce autophagy through the PERK-eIF2α-ATF4 and IRE1-ASK1-JNK1 pathways [32, 33]. The hyper-expression of death-associated protein kinase (DAPK) and DAPK related protein kinase (DRP-1) trigger cell membrane blebbing and extend autophagy continually [34]. DNA damage promotes autophagy through p53 signal [35]. Raf/MEK/ERK pathways stimulate autophagy in amino acid depletion condition [36]. Hypoxia irritates autophagy by up-regulation of hypoxia inducible factor-1(HIF-1) [37]. Together, these signaling pathways not only control autophagy but also are involved in cancerogenesis, and modulation of autophagy by targeting these pathways may affect autophagy-associated adaptive immune responses.

## AUTOPHAGY AFFECTS ADAPTIVE IMMUNITY

Recent accumulating evidences have shown that autophagy is also related to regulation of innate and adaptive immunity. Immune system utilized autophagy as an instrument to detect invading pathogens or monitor transforms in the status of self [38, 39]. Specific roles of autophagy in innate immunity, which is regulated by pathogen-recognition receptors (PRRs) signaling, include the regulation of the inflammasome and the clearance of apoptotic corpses to prevent either insufficient inflammatory or excessive inflammatory responses [17, 39]. In adaptive immunity, autophagy is essential to antigen presentation, thymus selection, lymphocyte development and homeostasis, which participates in anti-cancer effects. In this part, we therefore summarize current understanding of roles of autophagy in adaptive immune regulation.

### Autophagy in antigen presentation

T cells recognize intra- and extra-cellular antigen peptides that are presented to on major histocompatibility complex (MHC) molecules at cell surface, which is crucial for activation of CD4^+^ or CD8^+^ T cells, respectively. In generally, MHC-I molecules present antigenic peptides derived from intracellular proteins. For this purpose, MHC-I molecules are loaded mainly with proteasomal products for their recognition by CD8^+^ T cells, while MHC-II molecules receive antigenic peptides from extracellular antigens processed via lysosomal degradation for their recognition by CD4^+^ T cells [40]. However, there is an unconventional pathway named “cross-presentation”, which allows dendritic cells (DCs) to present extracellular antigenic peptides after lysosomal degradation through MHC-I molecules [41, 42]. Similarly, intracellular peptides can be loaded onto MHC-II molecules [43].

### Intracellular antigen processing for MHC class II presentation by autophagy

Autophagy can deliver cytoplasmic constituents for lysosomal hydrolysis, which contributes to the processing of intracellular antigens for presentation by MHC-II molecules. Some previous studies revealed that equal to 20% of natural MHC class II ligands are derived from cytosolic and nuclear proteins. Subsequent studies demonstrated that antigens including viral antigens, self-proteins and tumor antigens can be presented on MHC-II molecules [44, 45]. Biosynthesized, intracellular antigens presented by MHC-II also documented in B cells and fibroblasts. Brazil *et al*. demonstrated that presentation of endogenous C5 protein (a component of complement) by macrophage could be achieved when the macrophage were treated with low doses of the lysosome tropic agent ammonium choride, whereas in the presence of an inhibitor of autophagy presentation of biosynthesized C5 was inhibited [46]. In another *in vitro* experiment, the agents specifically blocking autophagy (3-MA and wortmannin) had been shown to reduce the capacity of DCs to present MHC-II-restricted peptide derived from endogenously synthesized mucin 1 protein(MUC1) [47]. Because MUC1 is a heterodimeric protein that is aberrantly expressed in various cancer cells including acute myeloid leukemia (AML) blasts and AML stem cells [48, 49], it is likely that autophagy induced by chemotherapic drugs and small molecular inhibitors in AML enhances the loading of MUC1 onto MHC molecules. Presentation by MHC molecules of peptides that suffer post-translational modifications and may form neo-antigens is a key mechanism for the activation of T cells. Autophagy in antigen presenting cells (APCs) has been demonstrated to result in presentation of citrullinated peptides to CD4^+^ T cells, which can be reduced by either 3-MA or ATG5 siRNA [50].

In addition to self-proteins, pathogen derived antigens including some viral and bacterial antigens that escape after endocytosis or release into the cytosol could also get processed via autophagy for MHC class II presentation. For example, Epstein Barr virus nuclear antigen 1(EBNA1) was found in autophagosomes, which could be presented to CD4^+^ T cells by EBV transformed B cells via MHC class II pathway, and Atg12 (an essential autophagy-inducing gene) siRNA inhibited recognition by EBNA-1-specific CD4^+^T cells [51]. However, there is a limited CD4 epitope display from endogenously expressed EBNA1 because autophagy is predominantly a cytoplasmic process [52].

**Figure 3:**
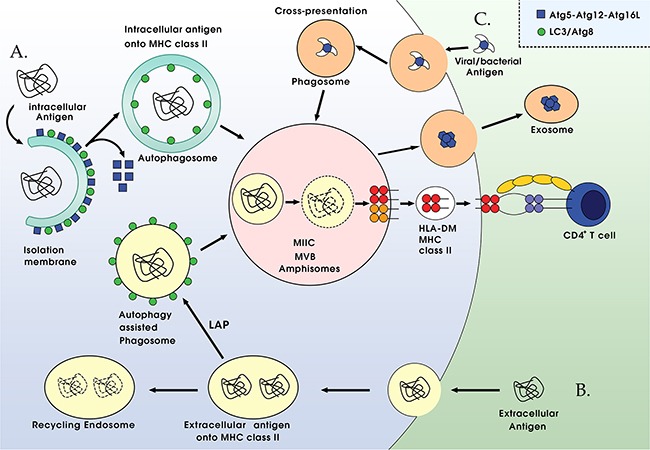
Autophagy-associated antigen presentation pathways **A**. Autophagy is a novel pathway for intracellular antigen presentation. Autophagosomes, which recruit cytosolic antigens to MHC class II containing compartments (MIICs) for lysosomal degradation and presentation to CD4^+^ T cells with the assistance of the peptide-loading chaperone HLA-DM. **B**. Extracelluar antigens, including phagosomes or apoptotic cells, get decorated with Atg8/LC3 (a process termed LAP), which enhances fusion with lysosomes, and might also increase fusion with MIICs for antigen loading onto MHC class II molecules. **C**. Autophagy machinery seem to be beneficial for cross-presentation on MHC class I molecules in the donor cell. Both viral and tumor antigens benefit from the cross-presentation via autophagy in the infected or transformed cells. The process of multivesicular bodies (MVBs) releases exosomes may be the key molecular machinery for cross-presentation.

Together, these reports suggest the important role of autophagy in intracellular antigen processing for MHC class II presentation to CD4^+^ T specific cells (Figure 3A).

### Extracellular antigen processing for MHC class II presentation by autophagy

Autophagy also plays an important role in facilitating the recognition of extracellular antigens phagocytesed by APCs in which antigens are delivered to autophagosomes [45, 53, 54]. For example, Atg5 and other proteins required for autophagy were demonstrated to be essential for optimal processing and presentation of a variety of forms of phagocytesed antigens containing Toll-like receptor (TLR) agonists [55]. It was reported that targeting of the Influenza Matrix Protein 1 (MP1) to autophagosomes via fusion to the Atg8/LC3 resulted in an enhanced MHC-II presentation to CD4^+^ T cells [44]. However, a more recent study showed that autophagy induced by influenza A virus failed to contribute to MHC-II-restricted presentation [56]. In addition to viral and bacterial antigen delivery for MHC II presentation after autophagy, another role for this catabolic process in tumor antigen delivery was recently suggested. Autophagic cargo that can be extruded into the extracellular matrix from cancer cells should be superior sources from which DCs can intake antigen for T cell priming [57].

Autophagy machinery contributes to deliver phagosomes to lysosomes for extracellular antigen processing [58]. However, the exact mechanisms of this process remain elusive. There are two hypotheses that have been put forward to explain enhanced phagosome processing with the help of autophagy: One is Atg8/LC3-associated phagocytosis (LAP), which recruited to phagosome membrane for strengthened fusion with lysosomes. And the other one is amphisomes formation, the procedure prior to lysosome fusion. Phagocytosis, a prominent endocytic pathway, has been found to be regulated by Atg proteins. During this LAP, LC3 seemed to be transiently recruited to a subset of phagosome membrane, which surrounded by pathogen-associated molecular pattern (PAMP) receptors, including the TLR family, primarily TLR2, or the C-type lectin Dectin-1, the T-cell immunoglobulin mucin protein 4 (TIM4) or Fc receptors for immunoglobulins, thus enhances phagosome fusing with lysosomes [58–60]. The generation of ROS produced by NADPH oxidases (NOX2) at the phagosome was proposed to be needed to maintain the conjugation of LC3 to phagosomes in LAP [61]. The fate of these phagosomes depends on cellular background. In some cell types, primarily mouse macrophages, the contents of LAP phagosomes seem to be degraded more rapidly than LC3 negative phagosomes, possibly because of more efficient transport along microtubules through LC3 binding to FYVE and coiled-coil domain containing 1 (FYCO1) protein, which accelerates LAP phagosomes fusing with lysosomes [62, 63]. Whereas, in plasmacytoid dendritic cells (pDCs) and human macrophages, LAP vesicles seem to be stabilized for fusion with TLR-containing endosomes and postponed the presentation of extracellular antigens for MHC class II [61]. Thus, the autophagy machinery that mediates LAP can affect the fate of phagosomes and facilitate the presentation of exogenous antigens by MHC class II (Figure 3B).

Amphisomes have been characterized and defined as an intermediate organelles, formed during autophagy through the fusion of endosomes and autophagosomes. Complex multi-vesicular vacuoles, an amphisome-like structure, have been observed in various cell types [64, 65]. But, whether amphisomeis an alternative for phagosomes fusion with autophagosomes for more efficient delivery of endocytosed cargo, or just a tentative structure with no biological effects is still enigmatic.

### Antigen packaging for cross-presentation via autophagy

Limited evidence displays that autophagy plays a role in the conventional MHC class I presentation, however, autophagy machinery has been implicated in the presentation of extracellular, endocytosed antigens by MHC class I molecules, a pathway termed cross-presentation that plays a critical role in cytotoxic T cell immunity against viruses and tumors. The autophagic exocytosis of antigen donor cell might benefit antigen processing and had been shown to facilitate the cross-presentation of tumor and viral antigens [66, 67]. Li, *et al*. demonstrated that autophagy in melanoma cells or ovalbumin antigen-expressing human HEK 293T cells is essential for cross-presentation by DCs both *in vitro* and *in vivo* [66]. Inhibition of autophagy by the RNA-interference (RNAi)-mediated depletion of Atg12 or Beclin-1abolished cross-presentation almost completely, whereas induction of autophagy by rapamycin or starvation dramatically enhanced cross-presentation of tumor antigens [66]. Recently, a working model has been established to explain antigen accumulation inside autophagosomes: when both proteasomes and lysosomes are inhibited, short-lived proteins, defective ribosomal products, likely component of the antigen pool in tumor cells, and misfolded proteins accumulate and form protein aggregates. This process then induces autophagy via p62 and Atg8/LC3 interaction [68]. Of note, further studies on mechanism of cross-presentation and therapeutic efficacy showed potent anti-tumor efficacy of the autophagosome-based DRibble (DRiPs-containing blebs) vaccine [69, 70].

But how do autophagosomes and their contents leave cells for cross-presentation is difficult to envision. One most possibility might be dependent on unconventional secretion of proteins, briefly, autophagosomes fuse with late endosomes to generate multi-vesicular bodies (MVBs), which could release exosomes, an immunogenic vesicles [69, 70]. Therefore, it is tempting to speculate that autophagy could facilitate exosomes release, and then antigens can be secreted by an unconventional pathway from MVBs that receive input from autophagosomes. Thus, autophagy might facilitate the packaging of antigens efficiently for cross-presentation on MHC I molecules (Figure 3C).

## AUTOPHAGY AND T CELL HOMEOSTASIS

**Figure 4:**
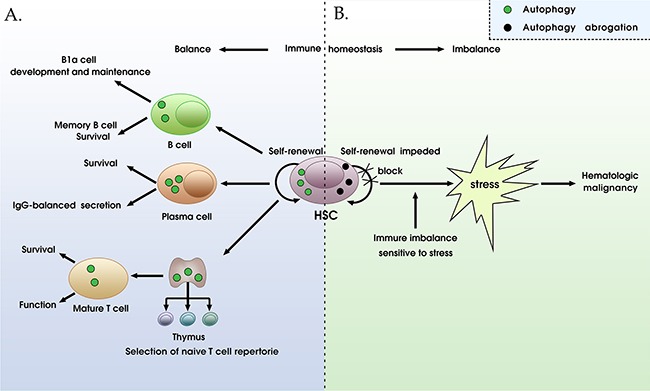
The roles of autophagy in immune homeostasis and tumorigenesis **A**. The normal state of autophagy regulation mechanism is crucial for immune homeostasis. Autophagy plays a crucial role in positive and negative selection of naïve T cells and is essential for mature T cell homeostasis and maintaining periphery T cell survival during proliferation. Autophagy also affects self-renewal of hematopoietic stem cells (HSCs), B1a cell development, memory B cell maintenance, plasma cell survival, and IgG-balanced secretion. **B**. The abrogation of autophagy leads to immune homeostasis imbalance, HSCs self-renewal impeded, cells are more sensitive to various stresses, eventually resulting in the initiation and progression of blood cancers including leukemia, myelodysplastic syndrome, and lymphoproliferative disorder.

In addition to antigen processing, autophagy modifies adaptive immunity *via* its contribution to the development, repertoire selection, maturation, homeostasis and effector functions of CD4^+^ / CD8^+^ T cells. Basal autophagy maintains homeostasis in T cells and can be up-regulated following T cell receptor (TCR) stimulation, and the absence of autophagy causes T cells differentiation abnormalities and function deficiency [71, 72]. Autophagy-associated presentation plays a crucial role in positive and negative selection of naïve T cells. When autophagy is abrogated, the number of naïve T cells drastically decreased [73–75]. Similarly, autophagy is also essential for mature T cell homeostasis and maintaining periphery T cell survival during proliferation, which entails clearance of damaged mitochondria and sustains proper Ca2^+^ homeostasis by trimming the ER [72–76]. The lack of autophagy results in increasing CD4^+^ T cell death, because of increased ROS production, elevated amounts of p38 (a mitogen-activated protein kinase), and imbalance of pro- and pre-apoptotic protein [77]. Moreover, deficiency in autophagy causes impaired survival of memory CD8^+^ T cells during infection with virus [78]. Furthermore, another role for autophagy in inducible natural killer T (iNKT) cells development has been displayed. The early stage of iNKT development is retarded in mice with deficiency of vps34/Beclin-1 [79]. Interestingly, autophagy has recently been found to modulate energy metabolism in T cells. With autophagy inhibitors treatment, ATP production might not be normally increased accompanying with the T cells activation, and ultimately, exhibit some defective in T cells, which could be reversed by exogenous energy source [80]. Thus, autophagy might be required for T-cell homeostasis and function, though much remains to be explored (Figure 4A).

## AUTOPHAGY IN THE DEVELOPMENT AND FUNCTION OF B CELLS

Current data from multiple studies have showed that autophagy plays a complex role in the development and function of B cells. Studies of Atg5 deficient mice revealed a key role for autophagy in B cell development and maintenance [81]. The quantity of B cells compromised when genes encoding essential Atg proteins are deficient, the decrease seems to originate from autophagy-deficient B-cell progenitors’ failure of transition between pro- and pre-B-cell stages in the bone marrow, suggesting autophagy is required for B cells development (Figure 4A).

The terminal step of the humoral immune response is attributed to plasma cells generate antibodies continuously. Besides, memory B cells can also be reactivated for antibody production upon encountering cognate antigen. Autophagy gene is essential for plasma cell homeostasis and the survival of memory B cells [82]. With the use of mice with B-cell-specific deletion of Atg5, Miller's group testified that plasma cell differentiation initiated by T cell-dependent/−independent antibody responses required autophagy [81]. Moreover, the long-term survival of plasma cells in the bone marrow are diminished in these autophagy deficient mice [83]. The absence of autophagy in plasma cells had a larger ER and more ER stress signaling than did their wild-type counterparts, which led to higher expression of the transcriptional repressor Blimp-1 and triggered unfolded protein responses during the production of immunoglobulins. The uncontrolled stress response was associated with less intracellular ATP and induced plasma cell death [84]. In addition to the maintenance of plasma cells, autophagy is also required for the survival of memory B cells. Mice with autophagy deficient B cells have deadly impaired specific secondary antibody responses to influenza A virus due to compromised maintenance of memory B cells [85].

Taken together, autophagy functions as an important role in B cells development, memory B cells survival and plasma cells homeostasis.

## THE ROLE OF AUTOPHAGY, AUTOPHAGY-ASSOCIATED ADAPTIVE IMMUNITY IN BLOOD CANCERS

Autophagy activity has been shown to be constitutively high in hematopoietic stem cells (HSC), and is required for the differentiation and self-renewal of HSCs [86–88]. It is well known that mechanisms protecting HSCs from cellular damage are essential to prevent hematopoietic malignancies. Autophagy is activated in response to various cellular stresses including DNA damage and genomic instability, removing unnecessary or harmful substances via lysosomal degradation mechinery [89, 90]. Several lines of evidence have shown that lack of autophagy in HSCs is involved in the pathogenesis of blood cancers [91, 92]. Beclin-1 haploinsufficiency contributes to the development of lymphoma and lymphoproliferative disease [91], whereas impairment of autophagy caused by Atg7 deletion leads to the expansion of progenitor cells in the bone marrow giving rise to a severe, invasive myeloproliferation [92]. Collectively, autophagy deficiencies impair HSCs, and cause oxidative stress, activation of the DNA damage response and genome instability, a known cause of blood cancer initiation and progression, which facilitates or even triggers tumorigenesis (Figure 4B).

On the other hand, when tumor is established, autophagy is able function as a pro-survival pathway. Indeed, cancer cells utilize autophagy as a means to adapt to the hypoxic, nutrient and growth factor deprivation, and metabolically stressful tumor microenvironment and therapeutically induced cell stress or damage [93]. Pharmacologic such as chloroquine and hydroxychloroquine approved by the U.S. Food and Drug administration for clinical use [94] or genetic inhibition of autophagy restores chemosensitivity and enhances tumor cell apoptosis in many types of hematopoietic malignancies [95]. However, autophagy also represents a distinct mechanism of cell death (autophagic cell death, ACD) in well-defined circumstances. For example, arsenic trioxide was showed to induce ACD in leukemic cell lines and AML progenitors, which could be reversed by knockdown of Beclin-1 or Atg7 [96]. We have reported that overexpression of Beclin-1 delivered by an oncolytic virus could induce significant ACD in a variety of leukemic cell lines and primary leukemic blasts [97]. ACD induced by anticancer agents in blood cancers has been summarized in an excellent review [95].

Recently, a deeper understanding of the process of immunogenic cell death (ICD) that can elicit a protective immune response against dead-tumor cell antigens induced by ICD inducers such as anticancer cytotoxic drugs and small molecular inhibitors has highlighted the importance of cancer immunotherapies and proposed novel antitumor strategies [98, 99]. There is increasing evidence to suggest some chemotherapeutic agents are intrinsically endowed with ability to trigger ICD (Table 1). These cytotoxic anticancer drugs are employed in the clinic for the treatment of hematologic malignancies, including various anthracyclines (such as doxorubicin, epirubicin, andidarubicin), mitoxantrone, oxaliplatin, cyclophosphamide, the histone deacetylase inhibitor (vorinostat) and bortezomib, a proteasomal inhibitor [99–101]. In AML cells, cytarabine, daunorubicin, all-trans retinoic acid (ATRA) and valproic acid were also found to induce increased calreticulin exposure (ecto-CRT) and release of HSP70 and HSP90, which indicating an induction of immunogenic apoptosis, although the level of CRT exposure/HSP release seems to depend on individual patients characteristics rather than the apoptosis-inducing drug [102]. Bortezomib had been showed to induce immunogenic death of human multiple myeloma, including primary tumor cells, which is dependent on cell-cell contact and linked to the expression of Hsp90 on the surface of dying cells [103]. More recently, diverse pro-apoptotic drugs, including topoisomerase II inhibitors, kinase inhibitors, and proteosome inhibitors have been shown to activate pannexin-1 channels and ATP release in Jurkat T cell acute lymphocytic leukemia model, which mediate immunogenic anti-tumor responses [104]. It is important to note that these anticancer agents could induce autophagy in both solid tumors and blood cancers (Table 1), thereby the relationship between autophagy in response to the cytotoxic agents and ICD in blood cancers deserve further attention.

**Table 1:** ICD-induced therapeutic compounds modulate autophagy in hematologic malignancies

Compounds	Bona fide ICD inducer	Molecular basis for ICD	Types of tumors (Ref.)	Effect on autophagy	Types of cancers(Ref.)
Bortezomib	Yes	CALR exposureType I IFN productionHMGB1 release	MMLymphoma[103, 114, 115]	inducer	B-ALL[116]
Bleomycin	Yes	CALR exposureATP secretionType I IFN productionHMGB1 release	MelanomaColon carcinoma[117]	inducer	HL[117]
Cyclophosphamide	Yes	CALR exposureATP secretionType I IFN productionHMGB1 release	Lymphoma[119, 120]	inducer	B-cell lymphoma[121]
Doxorubicin	Yes	CALR exposureATP secretionType I IFN productionHMGB1 release	Colon cancerALLOvarian cancerProstate cancer[122–126]	inducer	MMB-cell lymphoma[121, 127]
Gemcitabine	No	ATP secretionHMGB1 release	Pancreatic ductal adenocarcinoma[128]	inducer	HL[129]
Idarubicin	Yes	CALR exposureHMGB1 release	Colon cancerALLOvarian cancerProstate cancer[123, 124, 130]	inducer	Leukemia[131]
Melphalan	n.d	HMGB1 release	LymphomaColorectal tumor[132]	inducer	MM[127]
Epirubicin	No	CALR exposureATP secretionHMGB1 release	n.a.[123, 124]	inducer	NHM[133, 134]
Mitoxantrone	Yes	CALR exposureATP secretionType I IFN productionHMGB1 release	Colon cancer[123, 135, 136]	n.a.	n.a.
Temozolomide	n.d.	ATP secretionHMGB1 release	Prostate cancerBreast cancer[137, 138]	inducer	NHM[139]
Cisplatin	No	ATP secretionHMGB1 release	Lung cancarColon carcinomaFibrosarcoma[101, 140–142]	inducer	NHM[143]
Oxaliplatin	Yes	CALR exposureATP secretionType I IFN production	Colon cancerColorectal cancer[135, 144, 145]	inducer	NHM[146]
Vorinostat	n.d.	CALR exposure	n.d.[99]	inducer	AMLMCL[147, 148]

Abbreviations: ICD, immunogenic cell death; CALR, calreticulin; HMGB1, high-mobility group box 1; ICD, IFN, interferon; MM, multiple myeloma; B-All, B cell acute lymphoblastic leukemia; HL, Hodgkin's lymphoma; AML, Acute myeloid leukemia; MCL, Mantle cell lymphoma; n.a., not applicable; n.d., not determined; NHM, non-hematologic malignancies.

A detailed discussion of the molecular and cellular mechanisms involved in chemotherapy induced ICD can be found in Ref [100, 105]. Briefly, ICD is preceded or accompanied by the emission by dying cancer cells of immunostimulatory molecules called damage-associated molecular patterns (DAMPs). DAMPs that are crucial for ICD consist of ATP, high-mobility group protein B1 (HMGB1), and exposed molecules on the outer membrane of dying cells such as calreticulin, heat-shock proteins (Hsp90 and Hsp70), and ER sessile proteins. Mounting evidence indicates that autophagy plays a critical role in the induction of ICD. Michaud *et al* [106]. reported that the process of autophagy is necessary for the anti-tumor immune response evoked by apoptotic tumor cells in response to chemotherapy by regulation of ATP release. HMGB1, a key DAMP factor, serve as powerful immunological adjuvants and mediates ICD in cancer therapy [107]. Autophagy regulates passive HMGB1 release from dying cells and active HMGB1 secretion [105, 108]. Additionally, ACD in cancer cells exhibits an ICD property, especially in the ICD induced by some cytotoxic agents (anthracyclines, mitoxantrone, and oxaliplatin), vorinostat and bortezomib [99]. However, there is a few studies suggest that autophagic response of melanoma cells to ER stress suppresses basal ecto-CRT and restrains ICD induced by photodynamic therapy, suggesting that the role of autophagy in ICD needs to be assessed in context with the cancer model and the type of ICD inducer [109, 110].

## CONCLUSIONS AND PERSPECTIVES

Autophagy, as an evolutionarily conserved catabolic process, has been implicated in regulation of various aspects of biological process, including cell survival/death, proliferation, differentiation, senescence, and carcinogenesis. Current evidence suggests an important role for autophagy in both innate and adaptive immunity. In fact, autophagy is widely involved in antigen processing, and presentation, or function of antigen donor cells, APCs and T cells. Furthermore, premortem autophagy has been to determine the immunogenicity of chemotherapy-induced cancer cell death via promoting release of ATP. Like apoptotic cell death, autophagic cell death also entails immunogenicity after anticancer treatments. As autophagy plays multiple roles in antitumor immune, it is a potential target for therapies in cancer including hematologic malignancy.

Dysregulation of autophagy in HSCs is linked to the initiation and progression of blood cancers including leukemia [111], myelodysplastic syndrome [112], and lymphoproliferative disorder [113]. In blood cancer cells, autophagy is also a double-edged sword. Recently, many clinical trials using autophagy inhibitor, such as chloroquine and its analogue hydroxychloroquine, are being applied to multiple blood cancer types, including acute myeloid leukemia, chronic myeloid leukemia and multiple myeloma, in combination with chemotherapies or target therapies (ClinicalTrials.gov ID: NCT02631252; NCT01227135; NCT01689987 and NCT00568880). However, an interesting unresolved is whether systemic autophagy inactivation will be sufficiently selective to kill cancer cells while sparing immune cells from the deleterious consequences. Although autophagy-triggered modulation of anticancer immune response is a very complicated process, we need to elucidate some aspects of antitumor immunity in the patients with hematologic malignancy when autophagy inhibitor is administered.
